# Illumina Sequencing Approach to Characterize Thiamine Metabolism Related Bacteria and the Impacts of Thiamine Supplementation on Ruminal Microbiota in Dairy Cows Fed High-Grain Diets

**DOI:** 10.3389/fmicb.2017.01818

**Published:** 2017-09-20

**Authors:** Xiaohua Pan, Fuguang Xue, Xuemei Nan, Zhiwen Tang, Kun Wang, Yves Beckers, Linshu Jiang, Benhai Xiong

**Affiliations:** ^1^State Key Laboratory of Animal Nutrition, Institute of Animal Science, Chinese Academy of Agricultural Sciences Beijing, China; ^2^Beijing Key Laboratory for Dairy Cow Nutrition, Beijing University of Agriculture Beijing, China; ^3^Precision Livestock and Nutrition, Gembloux Agro-Bio Tech, University of Liège Gembloux, Belgium

**Keywords:** high-grain feeding, bacterial community, thiamine, dairy cows, high-throughput sequencing

## Abstract

The requirements of thiamine in adult ruminants are mainly met by ruminal bacterial synthesis, and thiamine deficiencies will occur when dairy cows overfed with high grain diet. However, there is limited knowledge with regard to the ruminal thiamine synthesis bacteria, and whether thiamine deficiency is related to the altered bacterial community by high grain diet is still unclear. To explore thiamine synthesis bacteria and the response of ruminal microbiota to high grain feeding and thiamine supplementation, six rumen-cannulated Holstein cows were randomly assigned into a replicated 3 × 3 Latin square design trial. Three treatments were control diet (CON, 20% dietary starch, DM basis), high grain diet (HG, 33.2% dietary starch, DM basis) and high grain diet supplemented with 180 mg thiamine/kg DMI (HG+T). On day 21 of each period, rumen content samples were collected at 3 h postfeeding. Ruminal thiamine concentration was detected by high performance liquid chromatography. The microbiota composition was determined using Illumina MiSeq sequencing of 16S rRNA gene. Cows receiving thiamine supplementation had greater ruminal pH value, acetate and thiamine content in the rumen. Principal coordinate analysis and similarity analysis indicated that HG feeding and thiamine supplementation caused a strong shift in bacterial composition and structure in the rumen. At the genus level, compared with CON group, the relative abundances of 19 genera were significantly changed by HG feeding. Thiamine supplementation increased the abundance of cellulolytic bacteria including *Bacteroides, Ruminococcus 1, Pyramidobacter, Succinivibrio*, and *Ruminobacter*, and their increases enhanced the fiber degradation and ruminal acetate production in HG+T group. *Christensenellaceae R7, Lachnospira, Succiniclasticum*, and *Ruminococcaceae NK4A214* exhibited a negative response to thiamine supplementation. Moreover, correlation analysis revealed that ruminal thiamine concentration was positively correlated with *Bacteroides, Ruminococcus 1, Ruminobacter, Pyramidobacter*, and *Fibrobacter*. Taken together, we concluded that *Bacteroides, Ruminococcus 1, Ruminobacter, Pyramidobacter*, and *Fibrobacter* in rumen content may be associated with thiamine synthesis or thiamine is required for their growth and metabolism. In addition, thiamine supplementation can potentially improve rumen function, as indicated by greater numbers of cellulolytic bacteria within the rumen. These findings facilitate understanding of bacterial thiamine synthesis within rumen and thiamine's function in dairy cows.

## Introduction

In current intensive dairy production, dairy cows are often fed high grain diet or easily fermentable carbohydrates to maximize energy intake and support high milk production. However, overfeeding of high grain diet is associated with subacute ruminal acidosis (SARA), affects ruminal fermentation characteristics and the structure of bacterial community in rumen content (Khafipour et al., 2009a; Hook et al., 2011). Specifically, high grain feeding leads to a reduction in the abundances of cellulolytic bacteria (Fernando et al., 2010), an increase in the proportions of starch-fermenting and lactic acid producing bacteria (Khafipour et al., 2009a), and the enhanced lysis of gram-negative bacteria associated with increasing ruminal free lipopolysaccharide (LPS) (Khafipour et al., 2009b). Once LPS translocate from the gastrointestinal tract into the peripheral blood circulation, the host immune response will be triggered, causing metabolic alterations and inflammation (Gressley, 2014), which in turn greatly impact the production and health of dairy cows. Therefore, more attentions have been paid to prevent the occurrence of SARA, and the use of feed supplements such as yeast (AlZahal et al., 2014), dicarboxylic acids (Vyas et al., 2015) and sodium bicarbonate (Mao et al., 2016) have been suggested to enhance rumen microbial community and subsequently ruminal fermentation. Interestingly, our previous study found that thiamine supplementation may be a new strategy for SARA prevention, since thiamine supplementation could improve rumen fermentation by increasing ruminal pH value and acetate content, and decreasing ruminal lactate production in rumen fluid (Pan et al., 2016). Moreover, thiamine supplementation could also attenuate inflammation response by decreasing ruminal LPS levels and suppressing the expression of pro-inflammatory cytokines in rumen epithelium (Pan et al., 2017). To explore the mechanism of thiamine on ruminal fermentation, Wang et al. (2015) preliminarily studied the expression changes of several bacteria associated with lactate metabolism using real-time PCR technology. They found that thiamine supplementation balanced ruminal bacterial community by reducing the population of *S. bovis* and prompting the growth of *M. elsdenii*. Nonetheless, the richness, diversity and overall bacterial community's response to thiamine supplementation in dairy cows are still unclear.

On the other hand, the requirement of thiamine in adult ruminants is mainly met by ruminal microbial synthesis (Miller et al., 1986). However, thiamine deficiency will occur when sheep or cattle have high grain induced subacute or acute ruminal acidosis (Karapinar et al., 2010; Pan et al., 2016), and thiamine deficiency is associated with the increasing thiamine degradation by thiaminase (Brent, 1976) or the decreasing microbial thiamine synthesis activity. Up to now, except Silverman and Werkman (1939) reported that certain propionate producing bacteria could synthesize thiamine or its intermediates, little information is available about thiamine synthesis bacteria in the rumen. By far, high-throughput sequencing has been used to identify ureolytic bacterial community in the rumen (Jin et al., 2016) and key phylotypes for several metabolic disorders in humans and animals (Liang et al., 2014). Therefore, the aim of this study was to explore the key taxa associated with thiamine synthesis and reveal the microbiota response to thiamine supplementation under high grain diet by high-throughput sequencing.

## Materials and methods

### Animals, experimental design and dietary treatments

Animal care and procedures were in accordance with the Chinese guidelines for animal welfare and approved by Animal Care and Use Committee of the Chinese Academy of Agricultural Sciences.

Six Chinese Holstein dairy cows (627 ± 16.9 kg BW; 180 ± 6 DIM) in second-parity fitted with 10 cm ruminal cannulas (Bar Diamond, Parma, ID) were allocated to a replicated 3 × 3 Latin square design. Treatments included a control diet (CON; 20% starch, DM basis), a high grain diet (HG; 33.2% starch, DM basis), and high grain diet supplemented with 180 mg thiamine/kg DMI (HG+T). The diets were formulated according to NRC (2001) to meet or exceed the energy requirements of Holstein dairy cows yielding 20 kg of milk/d with 3.5% milk fat and 3.0% true protein. Details of ingredient analysis and chemical composition of dietary ingredients were given in Table S1. Cows were fed at 06:00 and 18:00 h, one-half of the allowed daily ration at each feeding, and thiamine (thiamine hydrochloride, purity ≥99%; Wanrong Science and Technology Development Co., Ltd., Wuhan, China) was administered via the rumen cannula twice daily after diets were supplied. Three periods were included, each experimental period consisted of 21 days, with a 14-day washout between periods, during which cows were offered CON diet. Throughout the whole experimental periods, the cows were housed in individual stalls and were allowed to fresh water freely during the trial.

### Rumen fluid sampling and parameters measurement

On day 21 of each period, approximately 500 ml of rumen contents were sampled from cranial, caudal, dorsal, and ventral aspects of the rumen at 3 h after morning feeding. Collected samples were strained through four layers of cheesecloth to obtain rumen fluid. The rumen fluid was divided into two aliquots. One aliquot (approximately 100 ml) was processed to analyze the pH value, the concentration of volatile fatty acids (VFA) and lactate as described by Pan et al. (2016). Thiamine concentration in rumen fluid was detected by high performance liquid chromatography according to Analytical Methods Committee (2000). The other aliquot (approximately 100 ml) was stored at −80°C immediately for microbial DNA extraction and further analysis.

### DNA extraction

Ruminal fluid samples were thawed at room temperature and a 1-mL aliquot was centrifuged at 10,000 × g for 1 min at 4°C and the supernatant was discarded. The pellet was used for DNA extraction using a QIAamp DNA Stool Mini Kit (Qiagen, Hilden, Germany) with the addition of a bead-beating step. Briefly, the pellet samples were homogenized with 0.5 g zirconium beads (0.5 mm in diameter) and 1.4 mL ASL buffer using a Mixer Mill MM 400 (Retsch, Haan, Germany) with vibrational frequency of 1,800 rpm and grinding time of 60 s at room temperature. Then the mixture was incubated at 70°C for 5 min to increase DNA yield. The supernatant was further processed using QIAamp kits according to the manufacturer's instructions. Extracted DNA was assessed by agarose gel (1%) electrophoresis and quantified using a NanoDrop spectrophotometer ND-1000 (Thermo Scientific, Waltham, MA, USA). DNA was stored at −80°C until further analysis.

### Sequencing, sequence processing and analysis

The bacteria 16S rRNA gene was amplified using the barcoded universal primers 338F (5′-barcorde-ACTCCTRCGGGAGGCAGCAG-3′) and 806R (5′-GGACTACCVGGGTATCTAAT-3′) spanning the V3–V4 hyper variable region (Ye et al., 2016), where barcode is an eight-base sequence unique to each sample. Polymerized chain reactions (PCR) were carried out in a triplicate 20 μL mixture containing 4 μL of 5× FastPfu Buffer, 2 μL of 2.5 mM dNTPs, 0.8 μL of each primer (5 μM), 0.4 μL of FastPfu Polymerase and 10 ng of template DNA. The thermal cycling conditions was 95°C for 3 min, followed by 27 cycles at 95°C for 30 s, 55°C for 30 s, and 72°C for 45 s and a final extension at 72°C for 10 min. Amplicons were extracted from 2% agarose gels and purified using the AxyPrep DNA Gel Extraction Kit (Axygen Biosciences, Union City, CA, USA) according to the manufacturer's instructions and quantified using QuantiFluor™-ST (Promega, USA). Amplicon libraries were generated using TruSeq™ DNA Sample Prep Kit (TransGen Biotech, China) following the manufacturer's recommendations. The paired-end sequenced (2 × 250 bp) was conducted on an Illumina MiSeq platform according to standard protocols (Caporaso et al., 2010).

Raw fastq files were demultiplexed, quality-filtered using QIIME (version 1.17) with the following criteria: (i) The 300 bp reads were truncated at any site receiving an average quality score <20 over a 50 bp sliding window, discarding the truncated reads that were shorter than 50 bp. (ii) Exact barcode matching, nucleotide mismatch in primer matching and reads containing ambiguous characters were removed. (iii) Only sequences that overlap longer than 10 bp were assembled according to their overlap sequence. Reads which could not be assembled were discarded. Operational Units (OTUs) were clustered with 97% similarity cutoff using UPARSE (Edgar et al., 2011) and chimeric sequences were identified and removed using UCHIME (Haas et al, 2011). Taxonomy was aligned by RDP classifier and compared with the SILVA (SILVA version 115) 16S rRNA database (Pruesse et al., 2007) using confidence threshold of 70% (Amato et al., 2013). Community diversity was estimated with the normalized reads using the based coverage estimator (ACE), Chao1, and Shannon indices. The unweighted UniFrac distance method (Lozupone et al., 2007) was used to perform a principal coordinates analysis (PCoA), and an analysis of similarity (ANOSIM) in QIIME with 999 permutations (R Core Team, 2013) was conducted to assess significant differences between samples.

### Statistical analysis

Data were checked for normal distribution and homogeneity by Shapiro-Wilk's and Levene's tests in SAS 9.2 (SAS Institute Inc., Cary, NC). Ruminal pH, VFA, thiamine content, bacterial abundance, and diversity index were analyzed using PROC MIXED of SAS 9.2 as shown in the following model: *Y*_*ijklm*_ = μ + *T*_*i*_ + *P*_*j*_ + *S*_*k*_ + *C*_*l*_(*S*_*k*_) + *O*_*m*_ + *T*_*i*_ × *P*_*j*_ + *T*_*i*_ × *S*_*k*_ + *e*_*ijklm*_, where *Y*_*ijklm*_ is the dependent variable, μ is the overall mean, *T*_*i*_ the fixed effect of treatment (*i* = 1–3), P_*j*_ is the fixed effect of period (*j* = 1–3), *S*_*k*_ is the random effect of Latin square (*k* = 1–2), *C*_*l*_(*S*_*k*_) is the random effect of cow nested in square (*l* = 1–6), *O*_*m*_ is the fixed carryover effect from the previous period (O = 0 if period = 1), *T*_*i*_ × *P*_*j*_ is the interaction of treatment and period, *T*_*i*_ × *S*_*k*_ is the interaction between treatment and Latin square replicate, and *e*_*ijklm*_ is the random residual error. In these analyses, the carryover effects, and the interactions between treatment and period or square weren't detected for all variables, and they were finally removed from the model. Significance was declared at *P* < 0.05 and a tendency was considered at 0.05 < *P* < 0.10.

Pearson correlations between bacterial communities and ruminal fermentation variables or thiamine content were assessed using the PROC CORR procedure of SAS 9.2. Only those bacterial taxa with an abundance ≥0.1% of the total community in at least one ruminal sample were included in the analysis. A correlation matrix was created and visualized in a heatmap format by the HemI software (Deng et al., 2014). The abundance of bacterial communities at the genus level and ruminal variables were considered to be correlated with each other when the correlation coefficients (r) were above 0.55 and *P* value below 0.05 (Wang et al., 2016).

### Nucleotide sequence accession number

All the raw sequences were submitted to the NCBI Sequence Read Archive (SRA; http://www.ncbi.nlm.nih.gov/Traces/sra/), under accession number SRP114812.

## Results

### Ruminal pH value and concentrations of thiamine, volatile fatty acids, and lactate

As shown in Table 1, high grain feeding decreased ruminal pH value, and the concentrations of thiamine and acetate in rumen fluid (*P* < 0.05), whereas the concentrations of propionate, isobutyrate, and lactate were increased significantly (*P* < 0.05) compared with CON cows. The changes in ruminal pH, lactate, thiamine and propionate caused by HG-diet were inversed by thiamine supplementation (*P* < 0.05). Butyrate, valerate, isovalerate and total VFA weren't affected by dietary treatments (*P* > 0.05).

**Table 1:** Effects of high grain feeding and thiamine supplementation on rumen fermentation parameters in dairy cows.

**Item**	**Experimental treatments**^1^			**SEM^2^**	***P*-value**
	**CON**	**HG**	**HG+T**		
Ruminal pH	6.35^a^	5.67^c^	6.06^b^	0.078	0.003
Thiamine (μg/L)	8.97^a^	2.89^c^	4.81^b^	0.23	<0.001
Lactate (mmol/L)	0.49^c^	1.81^a^	1.06^b^	0.079	<0.001
Acetate (mmol/L)	66.73^a^	59.89^b^	69.83^a^	2.17	0.027
Propionate (mmol/L)	22.63^c^	28.22^a^	24.81^b^	1.54	<0.001
Isobutyrate (mmol/L)	1.02^b^	1.55^a^	1.33^a^	0.083	0.006
Butyrate (mmol/L)	10.69	11.72	10.85	0.49	0.460
Isovalerate (mmol/L)	1.73	1.95	2.13	0.12	0.216
Valerate (mmol/L)	1.28	1.39	1.58	0.093	0.105
TVFA (mmol/L)^3^	104.08	104.72	110.53	2.5	0.142

a,b,c *Means within a row with different letters differ significantly (P < 0.05)*.

1 *CON, control diet; HG, high grain diet; HG+T, high grain diet supplemented with 180 mg thiamine/kg DMI*.

2 *SEM, standard error of the mean*.

3 *TVFA, total volatile fatty acid*.

### Diversity, richness and composition of bacterial communities in rumen fluid

**Figure 1:**
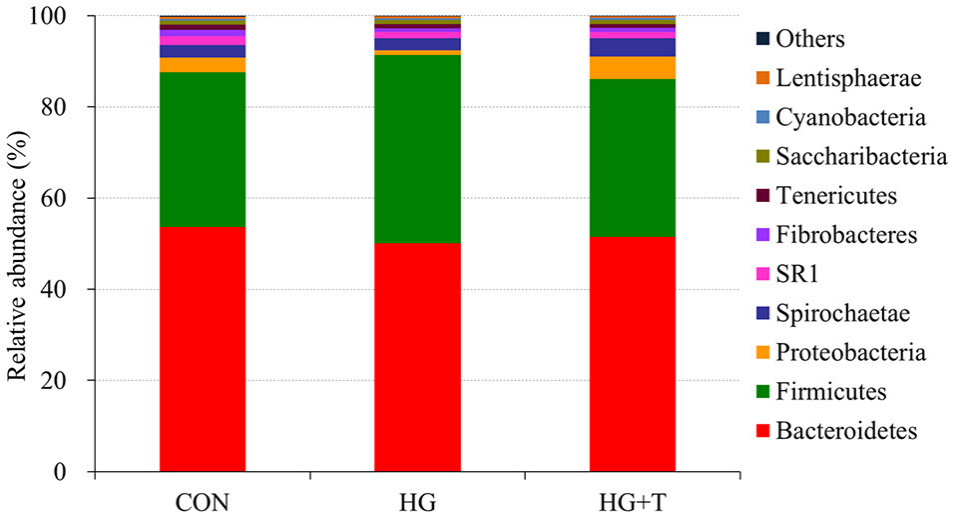
Percentage composition of the top 10 predominant phyla in rumen fluid.

Bacterial sequencing generated a total of 929,581 raw reads. Quality filtering at 97% similarity resulted in 697,247 high quality sequences, which clustered in 1,800 OTUs with 30,384 reads per sample after normalization, with an average of 1,374 ± 95 OTUs per sample. Within the bacterial population, 21 phyla were identified across all samples. *Bacteroidetes, Firmicutes*, and *Proteobacteria* were the three dominant phyla, representing 53.41, 35.10, and 3.07% of the total sequences, respectively (Figure 1). *Spirochaetae, SR1*, and *Fibrobacteres* represented average percentages of 2.91, 2.25, and 1.02%, respectively, of the total sequences. The proportion of some phyla (*Tenericutes, Saccharibacteria, Cyanobacteria, Lentisphaerae, Synergistetes*, and *Elusimicrobia*) was less than 1% of total microbial community, and other phyla such as *Actinobacteria, Fusobacteria, Chloroflexi* were not consistently present in all of the ruminal samples.

**Figure 2:**
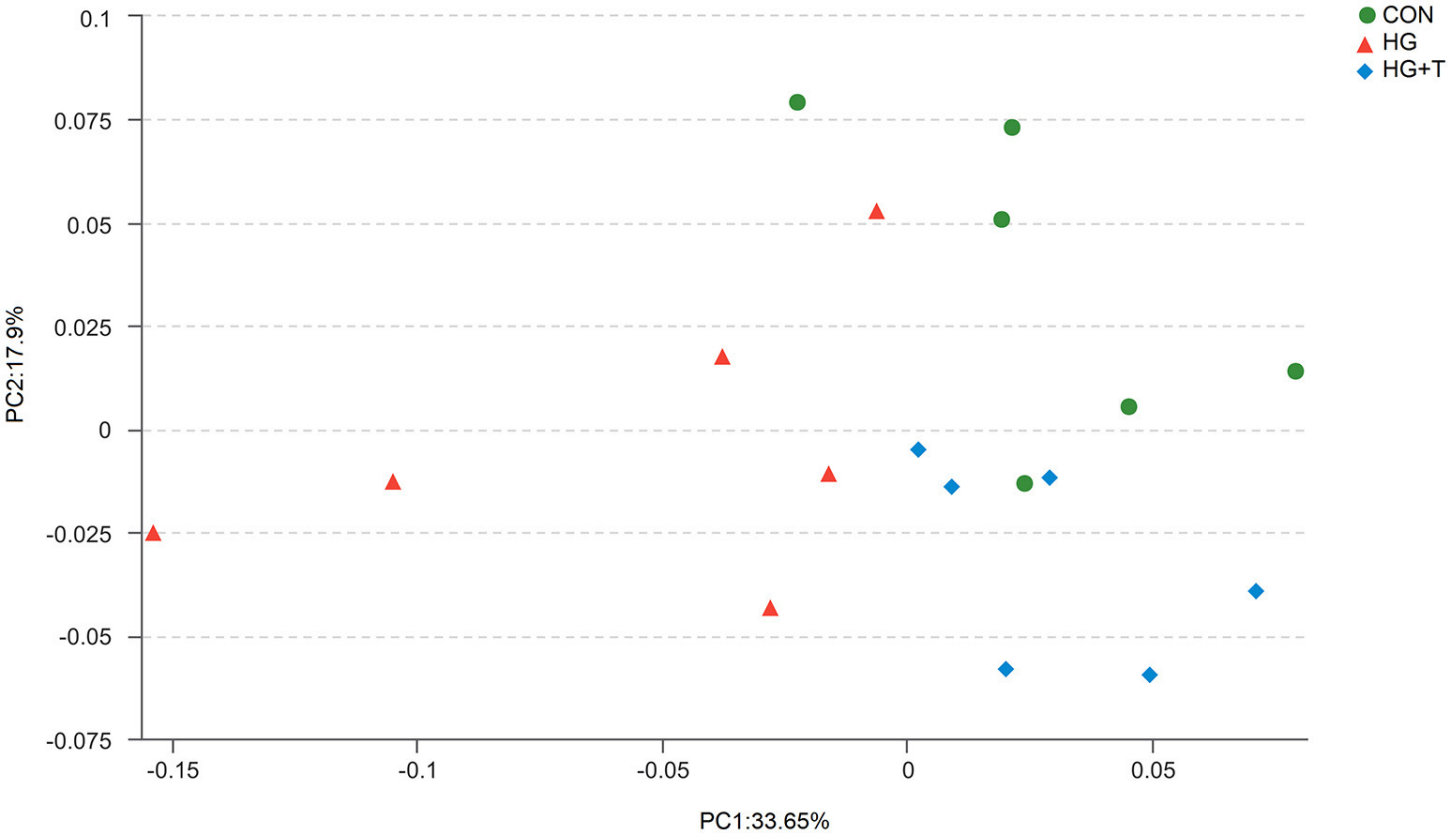
Principal coordinate analysis (PCoA) of bacterial community structures of the ruminal microbiota in CON (green circles), HG (red triangle), and HG+T (blue diamond) groups. PCoA plots were constructed using the unweighted UniFrac method. CON, control diet; HG, high grain diet; HG+T, high grain diet supplemented with 180 mg thiamine/kg DMI.

In terms of alpha bacterial diversity (Table 2), no differences were observed across treatments for the OTU numbers or Good's coverage, indicating that the sequencing depth was comparable across treatments. High grain diet decreased the abundance-based coverage estimator (ACE), Chao 1 richness and Shannon diversity index (*P* < 0.05) compared with CON diet. Thiamine supplementation had no significant effect on alpha bacterial diversity in respect to HG diet (*P* > 0.05). Principal coordinates analysis (PCoA) plots based on unweighted UniFrac distance metrics were conducted to compare the three treatments. The PCoA result exhibited that cows fed with HG diet were distinctly separated from cows in the CON and HG+T groups (Figure 2). The further ANOSIM analysis revealed a strong effect of high grain feeding on the structure of the bacterial community (*R* = 0.35, *P* = 0.01); Significant difference in bacterial community composition between HG and HG+T group was observed (*R* = 0.25, *P* = 0.02). Principal coordinate 1 and 2 accounted for 33.65 and 17.9% of the total variation, respectively.

**Table 2:** Number of observed species, richness and diversity indices in ruminal samples from each dietary treatment.

**Item**	**Experimental treatments**^1^			**SEM^2^**	***P*-value**
	**CON**	**HG**	**HG+T**		
OTU^3^	1,437	1,357	1,363	22	0.093
Good's coverage	0.99	0.99	0.99	0.0002	0.219
Chao1	1,576^a^	1,484^b^	1,519^a^^b^	21.8	0.048
ACE^4^	1,595^a^	1,492^b^	1,565^a^^b^	22.5	0.047
Shannon	5.99^a^	5.76^b^	5.75^b^	0.081	0.037
Simpson	0.010	0.015	0.013	0.004	0.266

a,b *Means within a row with different letters differ significantly (P < 0.05)*.

1 *CON, control diet; HG, high grain diet; HG+T, high grain diet supplemented with 180 mg thiamine/kg DMI*.

2 *SEM, standard error of the mean*.

3 *OTU, operational taxonomic units*.

4 *ACE, abundance-based coverage estimator*.

### Effect of HG feeding and thiamine supplementation on relative abundance of bacterial communities

The effects of high grain feeding and thiamine supplementation on main phyla were illustrated in Figure S1. High grain feeding increased the abundance of *Firmicutes* and decreased the abundance of *Proteobacteria* compared with other two treatments (*P* < 0.05). The decrease in *Proteobacteria* phyla under HG treatment was reversed by thiamine supplementation (*P* < 0.05), and the abundance of *Spirochaetae* was increased by thiamine supplementation significantly (*P* < 0.05). The other predominate phyla including *Bacteroidetes, SR1* and *Fibrobacteres* weren't affected by high grain feeding and thiamine supplementation (*P* > 0.05).

At the genus level, a total of 218 taxa were examined. The taxa with a relative abundance of ≥0.1% in at least one sample were further analyzed and the influenced genera by treatments were listed in Table 3. A total of 26 genera were affected (*P* < 0.05) by treatments; 19 genera of these were changed by HG feeding in respect to CON diet, and 15 genera were affected by thiamine supplementation compared with HG group. Specifically, compared with CON group, HG feeding decreased (*P* < 0.05) the proportions of *Bacteroides, unclassified Prevotellaceae, unclassified Bacteroidetes, Erysipelotrichaceae UCG-004, Fibrobacter, uncultured Lachnospiraceae, Eubacterium ventriosum, Ruminococcus 1, Ruminiclostridium 5, Selenomonas 1, Acetobacter, Succinivibrionaceae UCG-002, unclassified Succinivibrionaceae, Succinivibrio, Ruminobacter*, and *Pyramidobacter*. On the contrary, the relative abundance of *Succiniclasticum* (8.89 vs. 12.45%), *Ruminococcaceae NK4A214* (1.71 vs. 2.73%), and *Lachnospira* (0.06 vs. 0.13%) were significantly increased in HG group (*P* < 0.05). The decrease in genera of family *Succinivibrionaceae* and *Bacteroidetes, Ruminococcus 1* and *Pyramidobacter* caused by high grain feeding were reversed by thiamine supplementation (*P* < 0.05), and the proportion of *Treponema 2* was also significantly increased in HG+T group (2.74 vs. 4.22%; *P* < 0.05). Conversely, there was a significant decrease in relative abundance of *Succiniclasticum, Christensenellaceae R7, Ruminococcaceae NK4A214, Saccharofermentans* and *Papillibacter* (*P* < 0.05) associated with thiamine supplementation when compared with HG group.

**Table 3:** Effect of high grain feeding and thiamine supplementation on relative abundances of bacterial genera in rumen fluid using 16S rRNA sequencing^1^ (%).

**Phylum**	**Family**	**Genus/other**	**Experimental treatments**^2^			**SEM^3^**	***P*-value**
			**CON**	**HG**	**HG+T**		
*Bacteroidetes*	*Bacteroidaceae*	*Bacteroides*	0.44^a^	0.19^b^	0.30^a^	0.043	0.034
	*Prevotellaceae*	*Uncultured Prevotellaceae*	0.10^a^	0.06^b^	0.10^a^	0.018	0.022
	*Unclassified*	*Unclassified Bacteroidetes*	0.53^a^	0.45^b^	0.64^a^	0.027	0.008
*Firmicutes*	*Christensenellaceae*	*Christensenellaceae R7*	1.35^a^	2.02^a^	0.95^b^	0.21	0.011
	*Erysipelotrichaceae*	*Erysipelotrichaceae UCG-004*	0.21^a^	0.15^b^	0.13^b^	0.015	0.016
	*Fibrobacteraceae*	*Fibrobacter*	1.05^a^	0.60^b^	0.87^b^	0.081	0.046
	*Lachnospiraceae*	*Uncultured Lachnospiraceae*	0.79^a^	0.38^b^	0.32^b^	0.14	0.017
		*Eubacterium ventriosum*	0.13^a^	0.06^b^	0.08^b^	0.016	0.003
		*Oribacterium*	0.14^b^	0.15^b^	0.29^a^	0.023	0.011
		*Lachnospira*	0.06^b^	0.13^a^	0.16^a^	0.015	0.014
	*Ruminococcaceae*	*Ruminococcaceae NK4A214*	1.71^b^	2.73^a^	1.52^b^	0.16	0.003
		*Ruminococcus 1*	0.93^a^	0.54^b^	0.76^a^	0.05	0.005
		*Ruminiclostridium 5*	0.46^a^	0.21^b^	0.14^b^	0.048	0.004
		*Saccharofermentans*	0.76^a^	0.65^a^	0.51^b^	0.06	0.035
		*Papillibacter*	0.54^a^	0.46^a^	0.31^b^	0.038	0.018
		*Anaerotruncus*	0.12^a^	0.10^a^^b^	0.07^b^	0.015	0.050
	*Veillonellaceae*	*Succiniclasticum*	8.89^b^	12.45^a^	9.61^b^	1.18	0.035
		*Schwartzia*	0.14^b^	0.18^b^	0.35^a^	0.026	0.004
		*Selenomonas 1*	0.65^a^	0.30^b^	0.49^a^^b^	0.075	0.077
*Proteobacteria*	*Acetobacteraceae*	*Acetobacter*	0.17^a^	0.01^b^	0.008^b^	0.023	0.001
	*Succinivibrionaceae*	*Succinivibrionaceae UCG-002*	1.62^b^	0.30^c^	3.13^a^	0.15	<0.001
		*Unclassified Succinivibrionaceae*	0.24^a^	0.08^b^	0.21^a^	0.069	0.016
		*Succinivibrio*	0.07^a^	0.02^b^	0.09^a^	0.009	0.003
		*Ruminobacter*	0.09^a^	0.02^b^	0.06^a^	0.01	0.002
*Spirochaetae*	*Spirochaetaceae*	*Treponema 2*	2.77^b^	2.74^b^	4.22^a^	0.14	0.002
*Synergistetes*	*Synergistaceae*	*Pyramidobacter*	0.18^a^	0.04^c^	0.11^b^	0.013	<0.001

a,b,c *Means values within a row with different letters differ significantly (P < 0.05)*.

1 *Only bacterial genera (accounted for ≥0.1% in at least one of the samples) that affected by treatments were listed*.

2 *CON, control diet; HG, high grain diet; HG+T, high grain diet supplemented with 180 mg thiamine/kg DMI*.

3 *SEM, standard error of the mean*.

### Correlations between bacterial communities and ruminal variables

**Figure 3:**
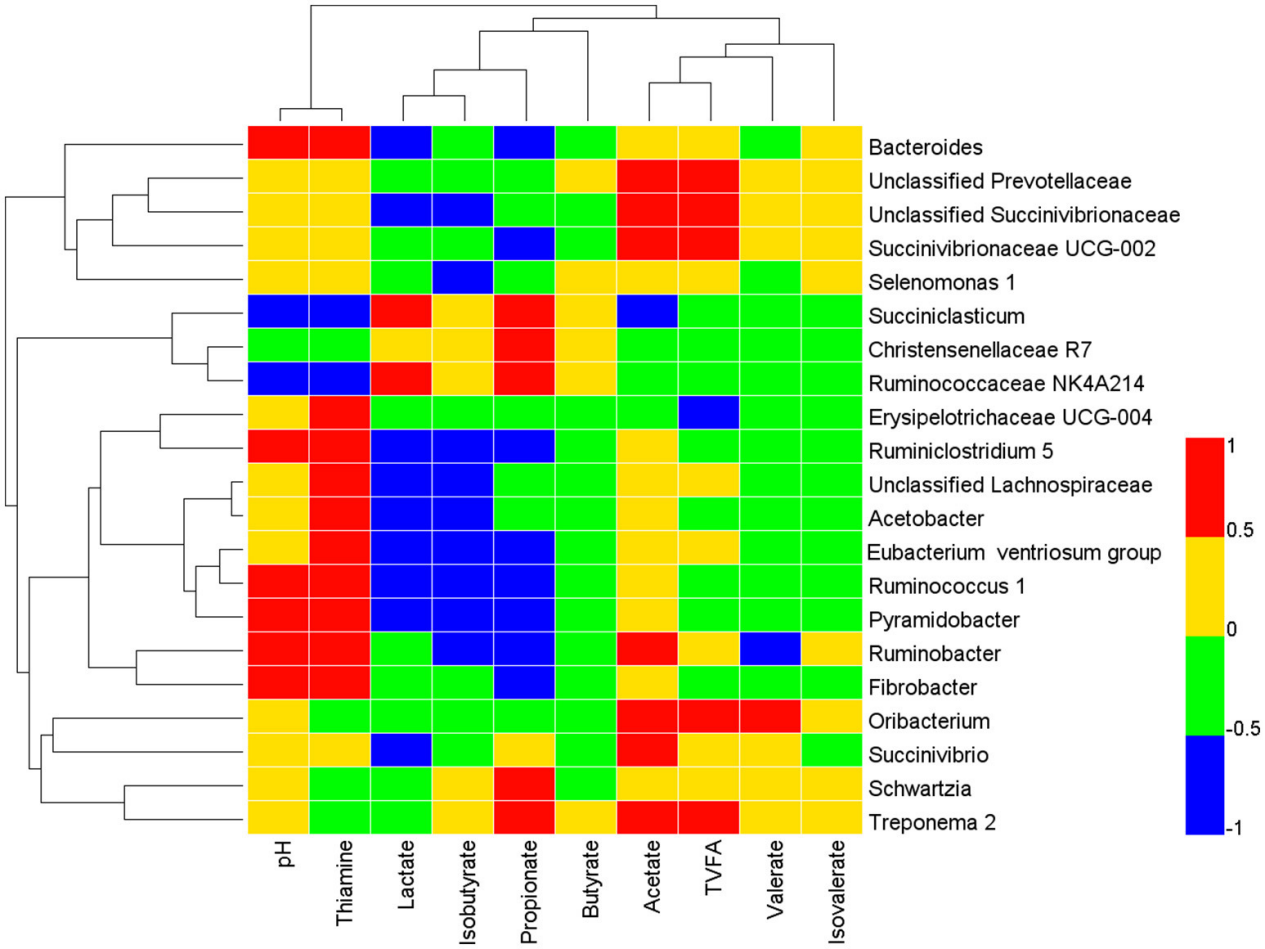
Correlation analyses between relative abundances of bacteria genera, and ruminal fermentation parameters and thiamine status. Only the genera with abundance significantly associated with the ruminal VFA concentration, thiamine concentrations, and pH were presented. The blue represents a negative correlation between the abundance of the species and the VFA concentration (*r* < −0.55, *P* < 0.05), the red color represents a positive correlation (*r* > 0.55, *P* < 0.05), and the green and yellow shows that the correlation was not significant (−0.55 < *r* < 0.55, *P* > 0.05).

As shown in Figure 3, the relative abundance of genera *Bacteroides, Ruminococcus 1, Ruminobacter, Pyramidobacter*, and *Fibrobacter* were positively correlated to ruminal pH and thiamine concentration (*r* > 0.55, *P* < 0.05), but negatively correlated to ruminal lactate content (*r* < −0.55, *P* < 0.05) except for *Ruminobacter* and *Fibrobacter*. Conversely, the genus *Succiniclastium* was negatively correlated with both ruminal pH and thiamine content (*r* < −0.55, *P* < 0.05), and positively correlated with lactate concentration (*r* > 0.55, *P* < 0.05). The acetate concentration was positively related (*P* < 0.05) with unclassified *Prevotellaceae* (*r* = 0.65), *Oribacterium* (*r* = 0.59), *Succinivibrionaceae UCG-002* (*r* = 0.73), unclassified *Succinivibrionaceae* (*r* = 0.68), *Succinivibrio* (*r* = 0.63), and *Ruminobacter* (*r* = 0.55), but negatively related with *Succiniclasticum* (*r* = −0.69). The relative abundance levels of 13 taxa were correlated (*P* < 0.05) with the ruminal propionate concentration [five positive (*Succiniclastium, Christensenellaceae R7, Schwartzia, Treponema 2, Ruminococcaceae NK4A214*) and eight negative (*Bacteroides, Eubacterium ventriosum group, Ruminococcus 1, Ruminiclostridium 5, Succinivibrionaceae UCG-002, Ruminobacter, Pyramidobacter, Fibrobacter*)]. The concentration of isobutyrate was negatively correlated (*P* < 0.05) with *Eubacterium ventriosum group* (*r* = −0.60), *Ruminococcus 1* (*r* = −0.69), *Acetobacter* (*r* = −0.76), *unclassified Succinivibrionaceae* (*r* = −0.59), and *Ruminobacter* (*r* = −0.61). No significant correlations were found (|*r*| < 0.55, *P* > 0.05) between the relative abundance of genera and butyrate, isovalerate, or total VFA.

## Discussion

### Effects of thiamine supplementation on ruminal microbiota composition under high grain feeding

In this study, we found that thiamine supplementation in HG diet could help improve rumen fermentation through increasing ruminal pH and acetate content, and reducing the accumulation of lactate in the rumen (Table 1). The improved rumen environment is mainly attributed to the stabilized ruminal microbial community (Pinloche et al., 2013). To understand the underlying mechanism of thiamine on rumen fermentation, the response of bacterial community to thiamine supplementation under high grain diet were investigated using high-throughput sequencing.

At phyla level, the high grain fed cows had higher abundance of *Firmicutes* and lower *Proteobacteria* compared with CON cows, since *Proteobacteria* are sensitive toward low pH and *Firmicutes* can still degrade easily fermentable carbohydrates under low pH (Wetzels et al., 2015). While the phyla changes above were reversed by thiamine supplementation, indicating thiamine could stabilize the bacterial community in the rumen. Besides, we found that thiamine supplementation significantly increased the relative abundance of *Spirochaetae* in respect to HG group. The possible reason is that most bacteria in *Spirochaetae* lack *de novo* thiamine biosynthesis pathway and need exogenous thiamine for growth (Bian et al., 2011).

At the genus levels, the thiamine treatment group had a higher proportion of the cellulolytic bacteria, including *Bacteroides, Ruminococcus 1 group, Succinivibrio* and *Pyramidobacter*, which may enhance the fiber degradation in the rumen. The possible reason for the enrichment of *Bacteroides* and *Ruminococcus 1 group* is that thiamine is an essential factor for their growth. Macy and Probst (1979) had certified that thiamine participated in the formation of α-ketoglutarate from succinic acid in *Bacteroides ruminicola*. Thiamine is essential for the growth of some strains in *Ruminococcus*, such as *Ruminococcus albus* (Bryant and Robinson, 1961) and *Ruminococcus flavefasciens* (Ungerfeld et al., 2009). *Succinivibrio* could promote the digestion of cellulose and hemicellulose (Sun et al., 2016), the increase of *Succinivibrio* may be caused by the increasing ruminal pH with thiamine supplementation, since *Succinivibrio* is positively correlated to the ruminal pH (Wetzels et al., 2016). Besides, *Pyramidobacter* species are vital cellulolytic bacteria and produce acetate as the main fermentation product (Bainbridge et al., 2016), the enhanced *Pyramidobacter* by thiamine could support fiber degradation, which explained the increasing ruminal acetate content in HG+T group. Moreover, *Selenomonas* 1 *group* in HG-fed cows tended to be stimulated by thiamine supplementation (*P* = 0.13). As a result, the increasing *Selenomonas* promoted the degradation of lactate from high starch diet, and thus helped to increase ruminal pH and prevented the occurrence of SARA in cattle. Above all, thiamine supplementation stimulated the growth of cellulolytic and lactate utilizing bacteria, thus increased ruminal pH and acetate production and subsequently improved rumen fermentation.

As the primary succinate utilizing bacteria, *Succiniclasticum* accounted for 12.45% of total bacterial community in HG group and increased significantly compared with CON group. The increase of *Succiniclasticum* was associated with the more production of succinate from starch degradation (Hook et al., 2011). Interestingly, thiamine supplementation reduced the proportions of *Succiniclasticum*. The possible reason was that thiamine, as the essential cofactor of pyruvate decarboxylation, stimulated the decarboxylation of pyruvate to produce acetate (Rastogi, 2010), thus reduced the production of succinate and subsequently decreased the proportion of *Succiniclasticum* in rumen content. In addition, thiamine supplementation decreased the abundance of *Christensenellaceae R7* and *Ruminococcaceae NK4A214*. However, little information about these two genera has been reported in the literature, the reasons for the altered status of genera by thiamine supplementation are unclear.

### Bacterial community associated with thiamine metabolism

As the coenzyme of pyruvate dehydrogenase and α-ketoglutarate dehydrogenase, thiamine plays a critical role in carbohydrate metabolism and is essential for normal cellular functions and growth (Said et al., 1999). The requirement of thiamine in dairy cows may enhance with the increasing carbohydrate levels in high grain diet. In the present study, the concentration of ruminal thiamine in HG-fed cows was lower than its content in CON cows (Table 1), which further proved that overfeeding high grain diet alters thiamine status (Pan et al., 2016). In order to explain the altered thiamine status during high grain feeding and to reveal possible bacteria related to thiamine metabolism, we conducted the correlation analysis between ruminal thiamine concentrations and microbial composition. The results showed that ruminal thiamine content was positively correlated with the genera *Bacteroides, Ruminococcus 1, Ruminobacter, Pyramidobacter*, and *Fibrobacter*, suggesting those genera may involve in thiamine synthesis, or thiamine is required for their growth and metabolism. Magnusdottir et al. (2015) found that *Firmicutes* can't synthesis thiamine monophosphate, and thiamine synthesis is most prevalent in *Bacteroidetes* and *Fusobacteria*. Silverman and Werkman (1939) reported that certain propionate—producing bacteria make thiamine or its intermediates, and Louis et al. (2014) pointed out that the main propionate production pathway is the succinate pathway, which is used by *Bacteroidetes* to generate propionate from carbohydrates. The literatures above proved that *Bacteroidetes* is highly related to thiamine synthesis, and thiamine biosynthesis gene has been identified in *Bacteroidetes fragilis* 638R (Veeranagouda et al., 2014). *Fibrobacter succinogenes*, as a major species in genus *Fibrobacter*, possess genes that encoded proteins involved in thiamine synthesis (Qi et al., 2008). For *Pyramidobacter*, the species *Pyramidobacter piscolens strain W5455* can synthesize thiamine by salvage pathway (BioCyc database; Caspi et al., 2015). Taken together, we deduced that *Bacteroidetes, Fibrobacter*, and *Pyramidobacter* play important roles in ruminal thiamine biosynthesis, and the altered thiamine status by high grain diet maybe related to the decreasing proportions of genera *Bacteroidetes, Fibrobacter*, and *Pyramidobacter* in the rumen.

Conversion of succinate to propionate was identified in genera *Succiniclasticum* (Van Gylswyk, 1995). During high grain feeding, *Succiniclasticum* increased to stabilize the rumen environment by degrading succinate to propionate (Hook et al., 2011; Liu et al., 2015). In our study, the increasing proportions of *Succiniclasticum* and decreasing thiamine concentrations leaded to their negative correlations, however, little information about thiamine effect on *Succiniclasticum* was reported in literature, their negative relationship is difficult to elucidate. The possible reason was that thiamine is the essential cofactor during pyruvate decarboxylation (Rastogi, 2010), the increasing thiamine content could promote the conversion of pyruvate to acetyl CoA, thus reduced the amount of succinate from pyruvate and subsequently the decreasing abundance of *Succiniclasticum*. Overall, alterations in bacterial populations under high grain feeding can interfere with thiamine synthesis in the rumen, and thiamine supplementation in HG diet helps to stabilize the structure of bacterial community in rumen fluid.

## Conclusion

In summary, high grain feeding had a profound impact on microbiota composition in rumen content. Thiamine supplementation to high grain diet improved rumen fermentation, which was partially attributed to the increasing abundances of *Bacteroides, Ruminococcus 1, Pyramidobacter, Succinivibrio*, and *Ruminobacter*, and the decreasing proportions of *Christensenellaceae R7, Lachnospira, Succiniclasticum*, and *Ruminococcaceae NK4A214*. Furthermore, ruminal thiamine content was closely related to the genera *Bacteroides, Ruminococcus 1, Ruminobacter*, and *Pyramidobacter*, these genera may participate in thiamine synthesis or thiamine is required for their growth and metabolism. Overall, our findings update understanding of thiamine synthesis in rumen bacteria and provide new strategies to improve dairy cows' health under high grain feeding pattern.

## Author contributions

XP, BX, and LJ designed the study. XP, FX, and ZT conducted the experiment. XP performed lab analysis and wrote the manuscript. FX, XN, and KW performed statistics and analyzed the data. XN, YB, and BX revised the paper. All authors carefully read and approved the final revision of the manuscript.

### Conflict of interest statement

The authors declare that the research was conducted in the absence of any commercial or financial relationships that could be construed as a potential conflict of interest.
