# Supplementary material for: Illumina Sequencing Approach to Characterize Thiamine Metabolism Related Bacteria and the Impacts of Thiamine Supplementation on Ruminal Microbiota in Dairy Cows Fed High-Grain Diets
Source: Front Microbiol. 2017 Sep 20;8:1818. doi: 10.3389/fmicb.2017.01818 (PMC5611408; doi:10.3389/fmicb.2017.01818)

Supplementary Material

Illumina sequencing approach to characterize thiamine metabolism related bacteria and the impacts of thiamine supplementation on ruminal microbiota in dairy cows fed high-grain diets

Xiaohua Pan, Fuguang Xue, Xuemei Nan, Zhiwen Tang, Kun Wang, Yves Beckers, Linshu Jiang and Benhai Xiong*

*** Correspondence:**

Benhai Xiong, State Key Laboratory of Animal Nutrition, Institute of Animal Science, Chinese Academy of Agricultural Sciences, Beijing 100193, China; xiongbenhai@caas.cn

Linshu Jiang, Beijing Key Laboratory for Dairy Cow Nutrition, Beijing University of Agriculture,

Beijing, 102206, China; jls@bac.edu.cn

1. Supplementary Figures and Tables

Table S1: Composition and nutrient levels of basal diets (Dry matter based)

Figure S1. Relative abundance of the main Bacterial phyla (>1%) in rumen fluid. (A) Bacteroidetes; (B) Firmicutes; (C) Proteobacteria; (D) Spirochaetae; (E) SR1; (F) Fibrobacteres. The top and bottom boundaries of each box indicate the 75th and 25th quartile values, respectively. The horizontal lines within each box represent the mean values.

Table S1. Ingredient and chemical composition of the experimental diets (Dry matter based) [1]

Items	CON	HG
Ingredients (*% of DM*)		
Chinese wildrye	11.0	5.0
Corn silage	34.0	15.0
Alfalfa hay	15.0	15.0
Ground corn	10.0	35.0
Soybean meal, 43% CP	14.0	14.0
Cottonseed meal	5.0	5.0
Distillers dried grains with solubles	5.0	5.0
Whole cottonseed	3.0	3.0
Limestone meal	1.0	1.0
Calcium hydrogen phosphate	0.7	0.7
Sodium chloride	0.5	0.5
Premix[2]	0.8	0.8
Nutrient composition (*% of DM*)		
DM (%)	46.87	50.53
NE_L[3], Mcal/kg	1.6	1.7
CP	18.2	18.0
Starch	20.0	33.2
NDF	36.2	25.8
ADF	23.4	17.7
NFC [4]	34.6	46.5
Ether extract	4.6	4.1
Ash	6.5	5.6
Calcium	0.88	0.84
Phosphorus	0.55	0.55
Thiamine (mg/kg)	1.7	2.3
DMI (kg/d)	15.8	16.6
Thiamine intake (mg/d)	26.9	38.2

[1] CON, control diet; HG, high grain diet.

[2] Premix contained (per kg): 1,350 mg of Cu, 2,240 mg of Mn, 4,320 mg of Zn, 32 mg of Co, 80 mg of I, 34 mg of Se, 480,000 IU of vitamin A, 100,000 IU of vitamin D, and 3,000 IU of vitamin E.

[3] NE_L was estimated according to NRC (2001).

[4] NFC = 100 – (% NDF + % CP + % Ether extract + % Ash) (NRC, 2001).

Figure S1. Relative abundance of the main Bacterial phyla (>1%) in rumen fluid. (A) Bacteroidetes; (B) Firmicutes; (C) Proteobacteria; (D) Spirochaetae; (E) SR1; (F) Fibrobacteres. The top and bottom boundaries of each box indicate the 75th and 25th quartile values, respectively. The horizontal lines within each box represent the mean values.

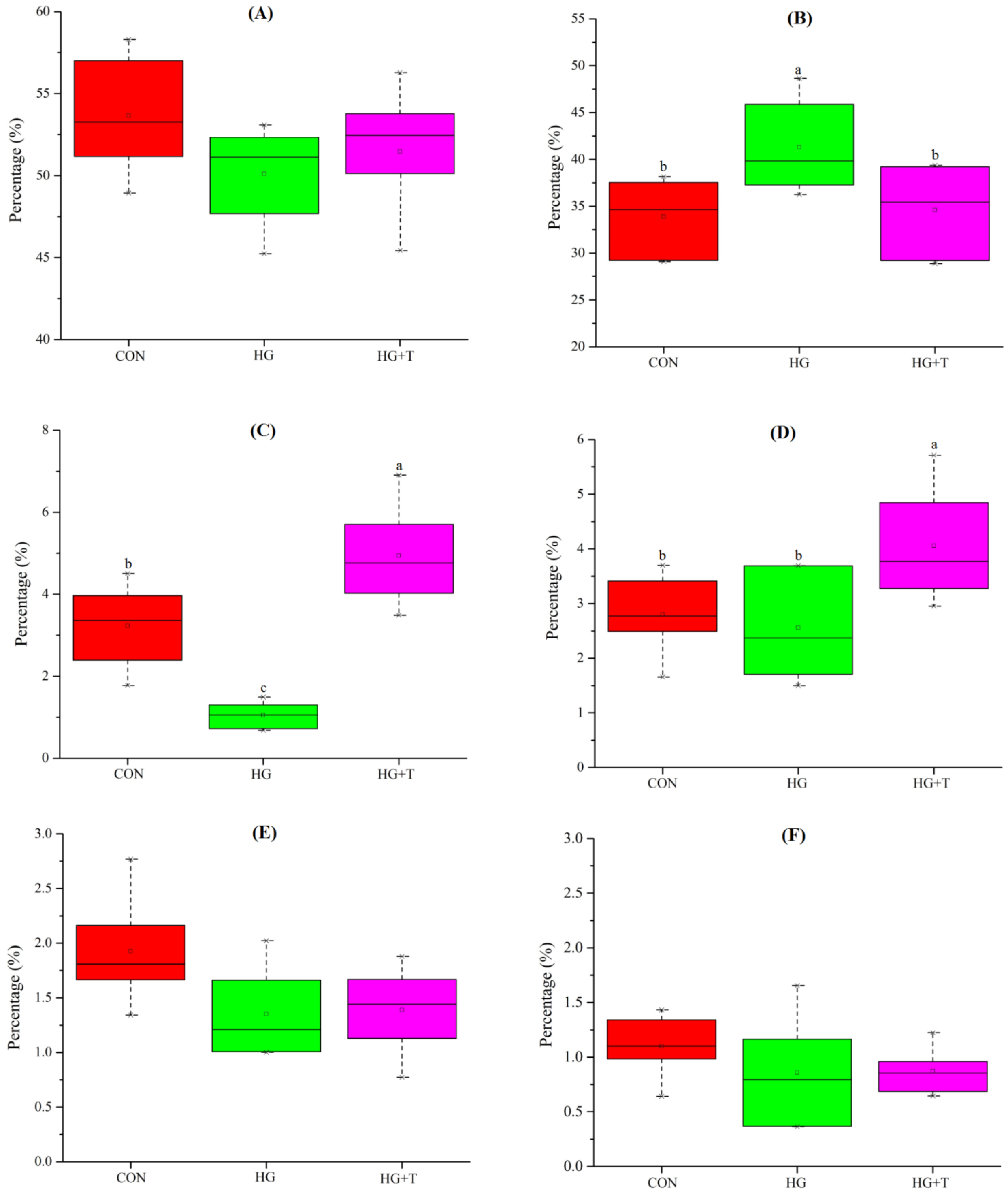

Supplement: Supplementary file 1 [file DataSheet1.PDF]
